# A Cost-Effective and Portable Optical Sensor System to Estimate Leaf Nitrogen and Water Contents in Crops

**DOI:** 10.3390/s20051449

**Published:** 2020-03-06

**Authors:** Mohammad Habibullah, Mohammad Reza Mohebian, Raju Soolanayakanahally, Khan A. Wahid, Anh Dinh

**Affiliations:** 1 Department of Electrical and Computer Engineering, University of Saskatchewan, Saskatoon, SK S7N 5A9, Canada; mr.mohebbian@usask.ca (M.R.M.); khan.wahid@usask.ca (K.A.W.); anv252@mail.usask.ca (A.D.); 2 Saskatoon Research and Development Centre, Agriculture and Agri-Food Canada, Saskatoon, SK S7N 0X2, Canada; raju.soolanayakanahally@canada.ca

**Keywords:** non-invasive, machine learning, leaf nitrogen, reflectance, plant phenotyping

## Abstract

Non-invasive determination of leaf nitrogen (N) and water contents is essential for ensuring the healthy growth of the plants. However, most of the existing methods to measure them are expensive. In this paper, a low-cost, portable multispectral sensor system is proposed to determine N and water contents in the leaves, non-invasively. Four different species of plants—canola, corn, soybean, and wheat—are used as test plants to investigate the utility of the proposed device. The sensor system comprises two multispectral sensors, visible (VIS) and near-infrared (NIR), detecting reflectance at 12 wavelengths (six from each sensor). Two separate experiments were performed in a controlled greenhouse environment, including N and water experiments. Spectral data were collected from 307 leaves (121 for N and 186 for water experiment), and the rational quadratic Gaussian process regression (GPR) algorithm was applied to correlate the reflectance data with actual N and water content. By performing five-fold cross-validation, the N estimation showed a coefficient of determination ($R^{2}$) of 63.91% for canola, 80.05% for corn, 82.29% for soybean, and 63.21% for wheat. For water content estimation, canola showed an $R^{2}$ of 18.02%, corn showed an $R^{2}$ of 68.41%, soybean showed an $R^{2}$ of 46.38%, and wheat showed an $R^{2}$ of 64.58%. The result reveals that the proposed low-cost sensor with an appropriate regression model can be used to determine N content. However, further investigation is needed to improve the water estimation results using the proposed device.

## 1. Introduction

Leaf nitrogen (N) determination in crop plants is crucial since it plays an essential role in plant growth and development, including photosynthesis and important enzyme production [1,2]. Application of N fertilizer at an optimum level for different growth stages is challenging, as the amount of N in soil is limited [3]. As a result, N fertilizer is often used excessively to achieve better yield and profitability [4]. However, most of the soil N content gets washed away by percolation [5]. On top of that, excessive N application affects the environment in several ways such as denitrification of soil, global warming, and water pollution [6]. Researchers are trying to discover methods to monitor plant N status over time. The current techniques include destructive and non-destructive approaches. Invasive determinations are basically chemical methods [7], namely, Kjeldahl digestion and Dumas combustion. There exist two approaches to determine plant N non-invasively, including light spectroscopy and hyperspectral imaging. The popularly used spectroscopic devices for sensing N are soil plant analysis development (SPAD) (Spectrum Technologies, Inc., Aurora, IL, USA) [8], atLEAF (FT Green LLC, Wilmington, DE, USA) [9], FieldSpec (Analytical Spectral Devices, Boulder, CO, USA) [10], GreenSeeker (Trimble, Sunnyvale, CA, USA) [11], and imagery from the QuickBird satellite (Ball Aerospace & Technologies, Boulder, CO, USA) [12]. Although SPAD, FieldSpec, GreenSeeker, and QuickBird imagery are widely used for N correlation, they have some limitations. For example, the basis of the SPAD meter and atLEAF involves determining chlorophyll, and it saturates at high N fertilization [13]. According to Xiong et al. (2015), the fraction of N assigned to chlorophyll is very small and most of it is allocated to photosynthetic proteins [13]. A good correlation ($R^{2}\;$= 0.86) with N using the FieldSpect 3 spectrometer was shown by Wang et al. [10]. However, this device is expensive and less flexible in terms of operating in the field. In addition, GreenSeeker is also expensive, and the determination saturates with increasing biomass/leaf area [6]. Moreover, the use of satellite imagery has some drawbacks such as satellite constant movement, cloudy weather, and subscription cost. Recently, hyperspectral imaging (HSI) was used in several plant phenotyping applications including N sensing [14]. This facilitates not only spectral information but also spatial information. Yu et al. (2014) showed how HSI can be used to investigate the mapping of N distribution in leaves [15]. However, HSI is normally used for research purposes as it is very expensive. Thus, developing a low-cost, quick, portable, lightweight device to determine leaf N concentration is very challenging.

The majority of plants take up N in the form of nitrate, although this depends on species. The chemical formation of N in plants is basically a protein having peptide bonds (–CO–NH–) [16], and these are sensitive to visible and near-infrared (NIR) regions. Blackmer et al. (1994) found that the reflectance at 550 nm wavelength is effective for the separation of different N treatments [17]. Many researchers developed NIR spectroscopy-based models to determine N in several plants including spring wheat (*Triticum aestivum* L., cv. “Bjarne”) [18], corn (*Zea mays* L.) [19], and winter oilseed rape (*Brassica napus* L.) [20]. In an article by Zhang et al. (2013), the authors published important wavelengths significant for detecting N using HSI in oilseed rape leaves, showing $R^{2}=0.882$ [21]. According to the authors, 12 optical bands around 440, 473, 513, 542, 659, 718, 744, 865, 928, 965, 986, and 1015 nm are effective for sensing N contents.

Leaf water content is another major factor for the overall health of the plants. One of the reasons is that water stress limits stomatal conductance and transpiration, affecting photosynthesis [22]. In addition, at a given crop growth stage, proper fertilizer application and irrigation management depend on leaf water content [23]. Thus, the determination of leaf water content is of great importance for monitoring the crop health status. One of the common methods of determining leaf water content is calculating the difference between fresh leaf weight and dried leaf weight. This method is destructive and time-consuming. However, the applications of remote sensing such as spectrometry and HSI were seen in several studies featuring non-destructive approaches. The determination of leaf water content in *Miscanthus* species (*M. sacchariflorus, M. sinensis,* and *M. fIoridulus*) by using visible/NIR wavelengths was investigated by Xioali et al. (2017). In that article, least-square support machine regression was used to model leaf water content with reflectance spectra [24]. They also identified 75 significant wavelengths between 450 and 2500 nm showing an $R^{2}\;\mathrm{of}\;\mathrm{about}\;0.9899.$ Moreover, Gente et al. (2013) utilized the terahertz time-domain spectroscopy technique to calculate the relative volumetric fraction of water present in the tissue, which correlates very well with the direct determination of water content [25]. The recent development of HSI proved to be very effective for in vivo analysis of plant chemical properties including water content [26]. In that study, Pandey et al. (2017) showed how HSI can be used to correlate hyperspectral images with leaf water content ($R^{2}=0.93$). In another study, where a UV–Vis spectrometer (Evolution 300) was used, it was shown that eight efficient wavelength intervals were effective for water content determination in leaves [27]. These wavelengths were 553–556 nm, 689–720 nm, 755–842 nm, 950–970 nm, 1013–1034 nm, and 1055–1075 nm. The main shortcoming of HIS and spectrometric techniques is that they are expensive.

The aim of this work is to develop a cost-effective, reflectance-based multispectral sensor (MS) that can estimate the actual N and water contents of leaves. To do so, we utilize a state-of-the-art machine learning algorithm to model the reflectance data captured from canola, corn, soybean, and wheat leaves at 12 wavelengths (450 nm, 500 nm, 550 nm, 570 nm, 600 nm, 610 nm, 650 nm, 680 nm, 730 nm, 760 nm, 810 nm, and 860 nm) in the visible (Vis)/NIR regions.

## 2. Materials and Methods

### 2.1. Designed Hardware Sensing System

The developed hardware prototype consisted of two optical sensors: Sensor1 and Sensor2. Sensor1 was a visible multispectral sensor (AS7262, AMS, Alvin, TX, USA), and Sensor2 is an NIR multispectral sensor (AS7263, AMS, Alvin, TX, USA). Moreover, a Qwiic mux breakout board (TCA9548A, SparkFun Electronics, Niwot, CO, USA) as a multiplexer (MUX), and a Raspberry Pi version 3 (RP3) as control circuitry were used. In addition, a power bank (BWA18WI035C, Blackweb, Bentonville, AR, USA) and an organic light-emitting diode (OLED) display (DS-OLED-MOD, Cytron Technologies, Pulau Pinang, Malaysia) were utilized in this prototype.

Sensor1 was a six-channel multispectral sensor in the visible range around 430 nm to 670 nm with full-width half-maximum (FWHM) of 40 nm. In this study, the visible AS7262 spectral breakout (SEN-14347, SparkFun Electronics, Niwot, CO, USA) was used, where Sensor1 was integrated. Here, it had built-in aperture controls of the light entering the process of the sensor array. Moreover, it had an I2C (Inter-Integrated Circuit) register set via which spectral data could be accessed. Here, the six visible channels were 450 nm (channel V), 500 nm (channel B), 550 nm (channel G), 570 nm (channel Y), 600 nm (channel O), and 650 nm (channel R). It has a 16-bit ADC (analog-to-digital converter). Moreover, its operating voltage ranged from 2.7 V to 3.6 V with the I2C interface. The package field of view of the sensor was ±20°. Calibration and measurements were made using diffused light. Each channel was tested with gain = 16× at ambient temperature (25 °C) under a 5700 K white LED test condition with an irradiance of ~600 μW∙${\mathrm{cm}}^{2}$ (300–1000 nm). The measurement unit of the channel was μW∙${\mathrm{cm}}^{2}$ with an accuracy of 12%. The energy at each channel was calculated with a ±40-nm bandwidth around the center wavelengths. A built-in excitation light source was used in this study. It was a 5700K white LED (L130-5780HE1400001, Lumileds, San Jose, CA, USA) with a color rendering index (CRI) of 80.

Sensor2 was a digital six-channel spectrometer in the NIR light region. The NIR AS7262 spectral breakout (SEN-14351, SparkFun Electronics, Niwot, CO, USA) was used, where Sensor2 was integrated. It had six independent optical filters whose spectral response was defined in the NIR wavelengths from approximately 600 nm to 870 nm with full-width half-maximum (FWHM) of 20 nm. The channels were 610 nm (channel R), 680 nm (channel S), 730 nm (channel T), 760 nm (channel U), 810 nm (channel V), and 860 nm (channel W). The light source used in the test condition was an incandescent light with an irradiance of ~1500 μW∙${\mathrm{cm}}^{2}\;$(300–1000 nm). Furthermore, the energy at each channel was calculated with a ±33-nm bandwidth around the center wavelengths. As an NIR source light, the onboard 2700K warm LED (L130-2790001400001, Lumileds, San Jose, CA, USA) was utilized with a CRI of 90. The configurations similar to those of Sensor1 are not mentioned. 

**Figure 1:**
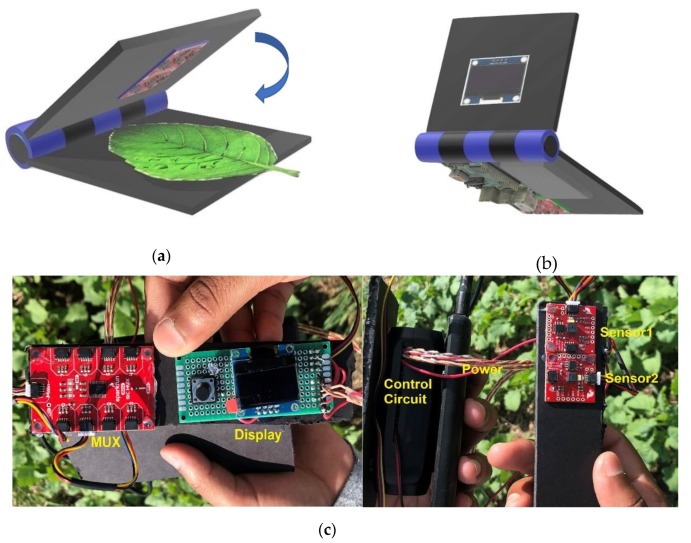
(**a**) The leaf is placed in between the clips; Sensor1 and Sensor2 touch the leaf during scanning. (**b**) Back view of the graphical set-up. Components of the sample device (**c**)—multiplexer (MUX), display, control circuit, power, Sensor1, and Sensor2.

RP3 (Raspberry Pi Foundation, Cambridge, UK) was used for controlling the sensors. The RP3 had an ARM processor (ARM Holdings, Cambridge, UK) having 1.2 GHz and 1 GB of LPDDR2 RAM (Low-Power Double Data Rate Synchronous Dynamic Random Access Memory). This widely popular board is used in several applications such as image processing [28] and IoT (Internet of Things) systems [29]. Both Sensor1 and Sensor2 had the same I2C address. Thus, a multiplexer (MUX) was used with eight configurable addresses of its own, providing 64 I2C buses. As a portable power supply for the device, a 5-V/2-A power bank with 5200 mAh was used. The prototype also included a 0.96-inch I2C OLED display for visualization. Overall, the device was organized in a clamp-like shape. Figure 1a,b shows the graphical set-up of the prototype, whereas Figure 1c shows the sample of the prototype. The leaf was placed as shown in Figure 1a; both sensors contact the leaf while scanning. Figure 1b shows the back view of the prototype.

**Figure 2:**
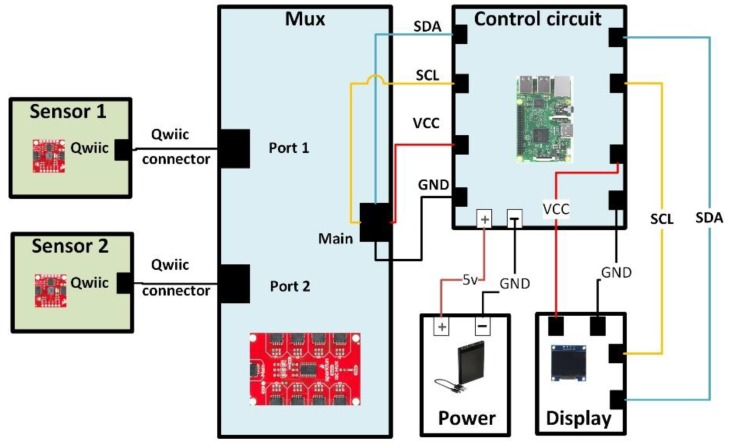
Connection overview of the proposed device. Sensor1 and Sensor2 were connected to Port1 and PORT2 of the MUX through Qwiic connectors. The four lines from the main port of the MUX were connected to the SDA, SCL, VCC, and GND of the control circuit. The power bank was connected to the micro-USB port of the control circuit, and the organic light-emitting diode (OLED) display was connected to the SDA and SCL.

Figure 2 shows the connection diagram of the prototype. Here, the Qwiic ports of Sensor1 and Sensor2 were connected to Port1 and Port2 of the MUX through Qwiic connectors. From the main port of the MUX, four wires SDA (Serial Data), SCL (Serial Clock), VCC (Voltage at Common Collector), and GND (Ground) were connected to pin numbers 3, 5, 1, and 9 of the control circuit, respectively. Moreover, the power bank was connected to the micro-USB (Universal Serial Bus) port of the control circuit, and the OLED display was connected to the SDA and SCL. Data collected from the sample leaves were modeled and processed using MATLAB 2018b. The overall set-up of the system is shown in Figure 2.

### 2.2. Greenhouse Experimental Set-Up

**Figure 3:**
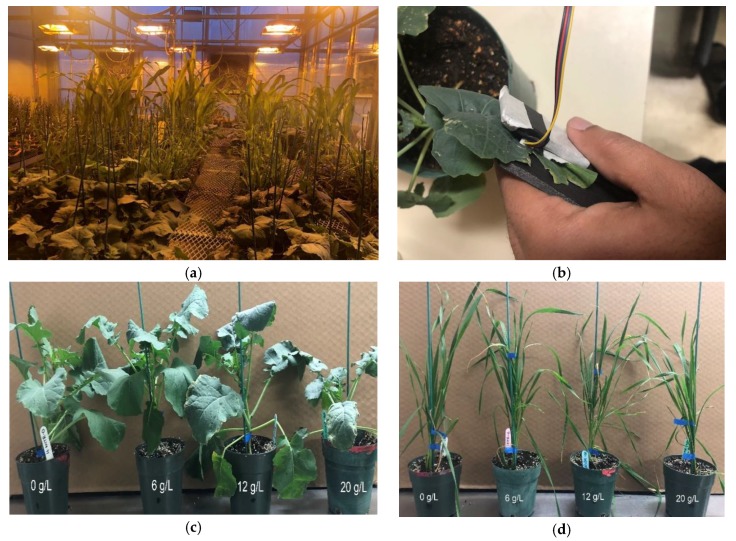

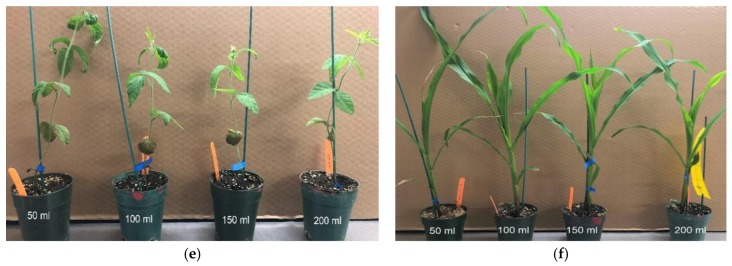
(**a**) Greenhouse-controlled environment. (**b**) Scanning the leaf using the proposed device. Sample pots of (**c**) canola and (**d**) wheat subjected to four levels (0 g∙L, 6 g∙L, 12 g∙L, and 20 g∙L) of N fertilization. Sample pots of (**e**) soybean and (**f**) corn subjected to four levels (50 mL, 100 mL, 150 mL, and 200 mL) of water application.

The experiment was conducted in two separate parts: an N experiment and a water experiment. The N experiment was conducted on a total of 64 plants consisting of commercially available canola (InVigor L140P), corn (P2702), soybean (S003-L3), and wheat (Stettler), with each having 16 pots. On the other hand, a water experiment was performed on separate 64 plants consisting of canola, corn, soybean, and wheat, with each having 16 pots. All the seeds were sown on 2 February 2019 in a controlled greenhouse environment situated at the Agriculture and Agri-Food Canada (AAFC), Saskatoon. The greenhouse conditions were set to 16 h day/8 h night, 23 °C day/ 18 °C night, 45% relative humidity. During the first three weeks, the plants were fertilized with slow-release 15–30–15 (15% nitrogen, 30% phosphorus, 15% potassium) fertilizer at a rate of 4 g∙L to ensure uniform establishment. Later, for the N experiment, the 64 pots were divided into four concentration levels ensuring equal distribution of plants from each species. Henceforward, only N fertilizer (30–0–0) was applied three times a week at four concentrations of 0 g∙L, 6 g∙L, 12 g∙L, and 20 g∙L. In the case of the water experiment, 64 pots were divided into four sections, with the same number of plants for each species for each section. After that, the water parameter was separated into four sections by applying it daily at a rate of 50 mL, 100 mL, 150 mL, and 200 mL. Figure 3a–f shows the greenhouse set-up and four different species of plants.

### 2.3. Spectral Data Collection and Ground Truth Measurement

**Figure 4:**
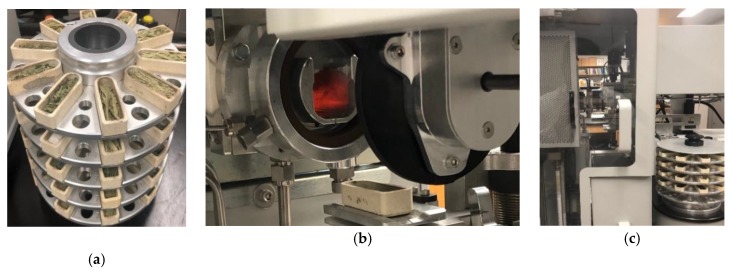
Actual N content measurement using an LECO TruMac N analyzer system (LECO Corporation, St. Joseph, MI, USA). Samples placed in the (**a**) stacked tray were collected automatically by a (**b**) combustion zone. (**c**) Overall set-up.

**Figure 5:**
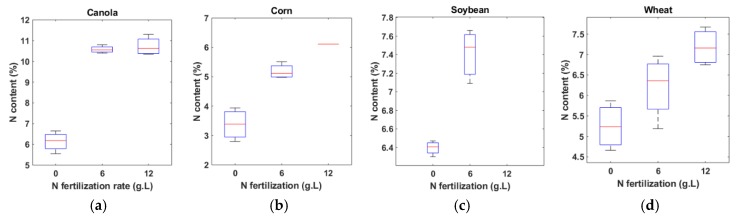
Boxplot of leaf N content of (**a**) canola, (**b**) corn, (**c**) soybean, and (**d**) wheat. Here, the horizontal axis represents the rate of N fertilization in g∙L, and the vertical axis represents the N content.

**Figure 6:**
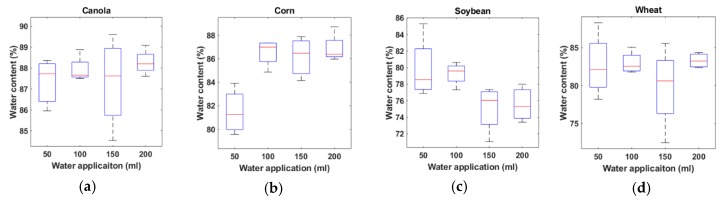
Boxplot of the leaf water content of (**a**) canola, (**b**) corn, (**c**) soybean, and (**d**) wheat. Here, the horizontal axis represents the rate of water application in mL, and the vertical axis represents the water content.

**Figure 7:**
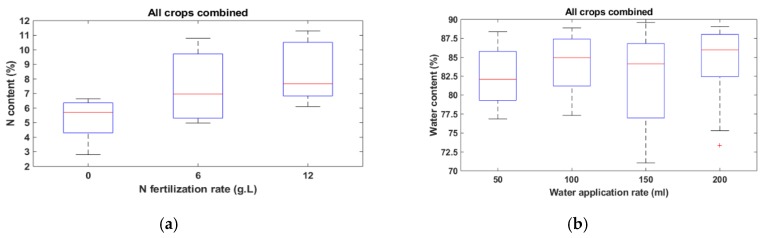
Boxplot of leaf (**a**) N content and (**b**) water content of all crops combined. The horizontal axis represents the rate of N fertilization in g∙L for (**a**) and water application in mL for (**b**), and the vertical axis represents the N content for (**a**) and water content for (**b**).

Data were collected each day from 19 March to 26 March 2019. At first, both sensors were calibrated each day by taking reflectance data from a white reflector. A regular white mirror paper [30] was used as the reflector. It is worthwhile mentioning that the same reflector was used all throughout the measurements to ensure the same reference. Reflectance at 12 wavelengths (450 nm, 500 nm, 550 nm, 570 nm, 600 nm, 610 nm, 650 nm, 680 nm, 730 nm, 760 nm, 810 nm, and 860 nm) was collected from the sample leaf surfaces one leaf at a time, with each taking 5 s to scan. In the case of the N experiment, the leaves from 20 g∙L fertilizer were not used due to toxicity resulting from over-fertilization. Furthermore, 12 g∙L samples of corn and soybean were not used for the same reason. Thus, the number of leaf samples used for this experiment was 121 including 36 canola, 28 corn, 21 soybean, and 36 wheat leaves. In the water experiment, one plant from corn and one plant from soybean were not used as they were dry at the time of data collection. Thus, a total of 186 leaves including 48 canola, 45 corn, 45 soybeans, and 48 wheat were used in the water experiment. For determining the actual water contents of the samples, the leaves were cut and then the fresh weights were measured ($W_{fresh}$). All the samples were then placed in an oven, dried at 50 °C for 72 to 96 h until completely dry. The dry weight of the samples was then obtained ($W_{dry}$). Leaf water content (WC) was calculated as $\mathrm{WC}=\left(W_{fresh}-W_{dry}\right)/W_{fresh}\;\times 100\;\%$. For measuring the actual N content of the dried samples, an LECO TruMac N analyzer was used as shown in Figure 4a–c. The analyzer is a macro combustion N degerminator that utilizes a pure oxygen environment in a ceramic boat for the macro sample combustion process. The box plots of N and water contents are shown in Figure 5, Figure 6 and Figure 7. The ranges of the N content for three levels of fertilizer application (0, 6, 12 g∙L) for canola, corn, and wheat, and two levels (0, 6 g∙L) of fertilizer for soybean are shown in Figure 5a–d. The variations of the 36 wheat samples were the most significant, followed by canola. The variations combining all the N samples are shown in Figure 7a. The lowest, mean, and highest N contents were found to be 2.8%, 6.8%, and 11.3%. The water content variations of the four species as a result of the different application rates are depicted in Figure 6a–d. Figure 7b shows the boxplots of the combined water contents. In these cases, the lowest, mean, and highest water contents were 71.04%, 83.16%, and 89.6%.

### 2.4. Data Preprocessing and Modeling

Feature selection approaches can be categorized into three categories including wrapper, filter, and embedded methods [31]. The filter methods work independently of the classifier. The wrapper feature selection methods formulate a problem, then search the problem space, before finding the best combination of features. Finally, the embedded method evaluates the accuracy of the classifier for predicting the best features with searching guided by a learning classification process. In our analysis, an independent-sample *t*-test, which is a filter method, was used to identify statistically discriminative normally distributed features [32]. Then, the normalization was applied to features to change the underlying probability distribution of features and to consequently improve the classification performance. A modified standard score (*z*-score) was used for normalization [33], wherein, instead of using an average, the median operator was implemented in order to make the normalization robust against outliers [34].

**Figure 8:**
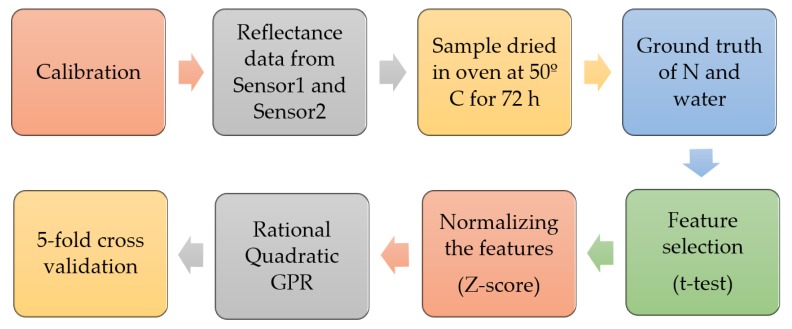
Process flow of the methodology.

For correlating the spectral data with the actual N content, several state-of-the-art machine learning regression algorithms were implemented. Among all of them, Gaussian process for regression (GPR) was found to be the most effective [35]. This method receives significant interest in statistical modeling for its good performance in prediction [36]. Gaussian process regression is a nonparametric, probabilistic, Bayesian approach based on kernels. This technique was used for classification [37] and regression [38] in different domains. In our regression analysis, GPR was used in both N and water experiments, for modeling the spectral data. “Rational quadratic” as the kernel and constant basis functions were specified in the GPR method. For validating the model on new data, k-fold cross-validation (CV) [39] was performed in this work using the value of k as five, i.e., dividing the dataset into five subsets. Each GPR was trained on four subsets and tested on the remaining set, and this was run five times to compute the average performance. Thus, every data point got the chance to be tested once and trained four times. The overall methodology is shown in Figure 8, as a process flow diagram.

### 2.5. Validation Metrics

The performance of the cross-validation model was evaluated in terms of primary metrics stated in Reference [40]. These were the root-mean-square error (RMSE), mean squared error (MSE), mean absolute error (MAE), and coefficient of determination ($R^{2}$). The mathematical definitions of the metrics are given in Table 1.

## 3. Results

### 3.1. Nitrogen Concentration Estimation 

**Figure 9:**
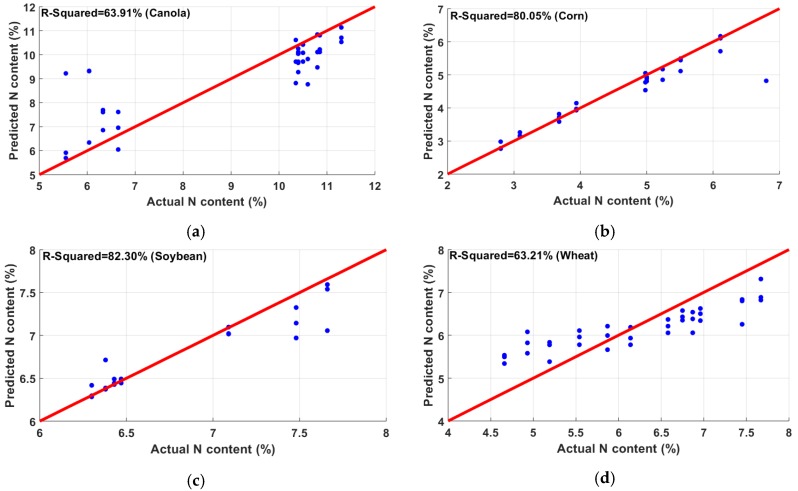
Correlation between the predicted N content and actual N content in (**a**) canola, (**b**) corn, (**c**) soybean, and (**d**) wheat. Soybean showed the best correlation of 82.3%, whereas wheat showed the lowest correlation (63.21%).

**Figure 10:**
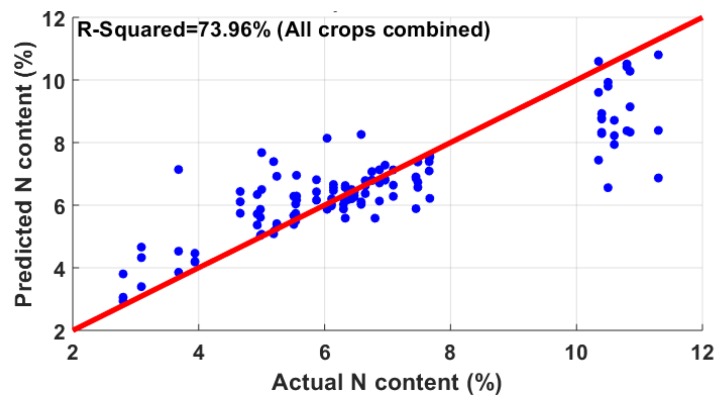
Nitrogen content estimation combining canola, corn, soybean, and wheat.

In all the analyses, a *t*-test was performed before regression modeling. With a 5% *p*-value, the *t*-test selected all 12 variables as important features. After normalizing, as discussed in the methodology section, GPR was used for correlating the reflectance data with crop measurements. In this section, the average results of five-fold cross-validation are reported. The regression was performed on individual species (Figure 9a–d), as well as their combination (Figure 10). In the N experiment, the best correlation was found in soybean ($R^{2}\;$= 82.29%) followed by corn ($R^{2}$ = 80.05%). Wheat showed the lowest correlation among all species, having an $R^{2}$ of 63.21%. The coefficient of determination shown by canola was 63.91%. Another regression analysis was performed combining all samples of the four species. The combined model showed an overall $R^{2}$ of 73.96% with an RMSE of 1.13, as shown in Figure 10; the summary of the results is shown in Table 2.

### 3.2. Water Content Estimation 

**Figure 11:**
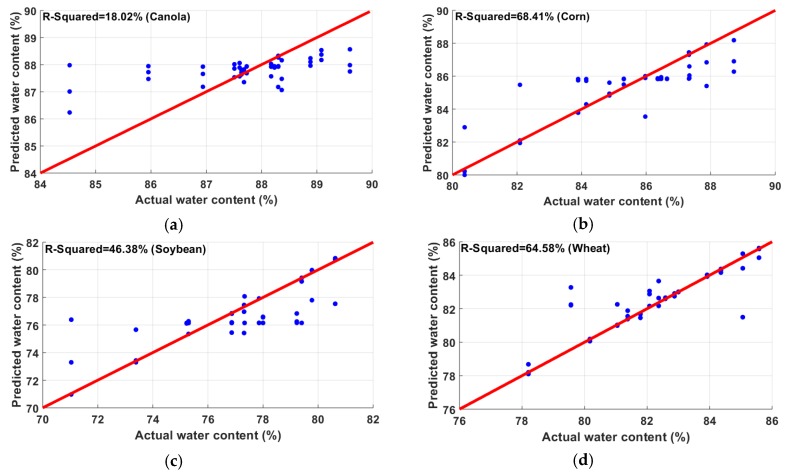
Correlation between the predicted water content and actual water content in (**a**) canola, (**b**) corn, (**c**) soybean, and (**d**) wheat. Corn showed the best correlation of 68.41%, while wheat showed a correlation of 64.58%; on the other hand, canola and soybean did not correlate well.

The five-fold cross-validation results of water content estimation are shown in Table 3. The correlation results reveal that the reflectance from canola did not correlate well with the leaf water content, having an $R^{2}$ of 18.02%. In the case of wheat, there were some outliers, which were outside the range of two standard deviations from the median. Those outliers were removed, with the sample then showing a correlation of 64.58%. The best correlation was observed in corn leaves (68.41%). Although the individual $R^{2}\;$ for canola and soybean was lower, a combination of canola and soybean showed a better estimation of 61.08%. The regression analysis combining corn, wheat, canola, and soybean showed an overall coefficient of determination of 46.08%. Thus, unlike the N correlation, the combined result for water estimation was not found to be satisfactory. Actual vs. predicted water content plots for the four species are shown in Figure 11a–d. 

### 3.3. Important Wavelengths

**Figure 12:**
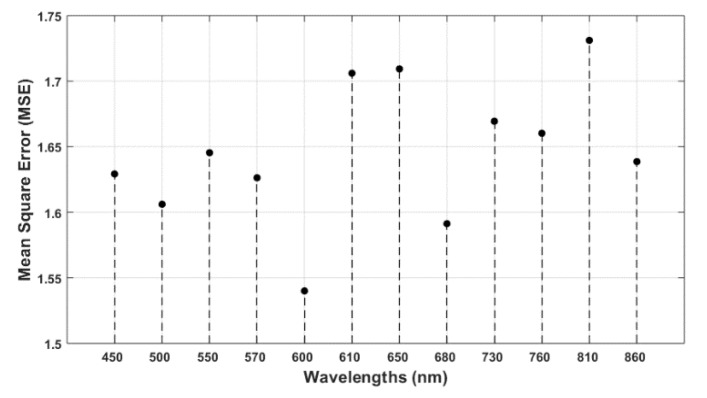
Important wavelengths for N are shown based on the increase in MSE. The three most significant wavelengths, in this analysis, were found to be 810 nm, 650 nm, and 610 nm.

**Figure 13:**
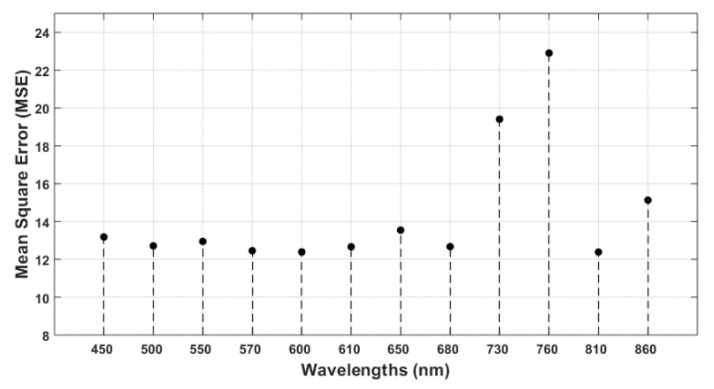
Important wavelengths for water are shown based on the increase in MSE. The most significant wavelength for water content determination was found to be 760 nm, followed by 730 nm and 860 nm.

In this section, the importance of the features (wavelengths) was determined by calculating the increase in MSE of five-fold cross-validation after each feature was removed. For example, in the case of N determination, when the feature of 810 nm was removed and the whole regression analysis was performed with the 11 other features, the average mean square error of 10 runs was 1.7311. On the other hand, for the 600 nm wavelength feature, the MSE was 1.54, which was much less than for 810 nm. This means that the decrease in correlation performance by removing the 810 nm wavelength as the feature was much more significant than that achieved by removing the 600 nm wavelength. Hence, reflectance at the 810 nm wavelength is more important than that at 600 nm with respect to determining N. After performing the same technique on 12 wavelengths individually, the importance of wavelengths was as shown in Figure 12. It was found that the three most significant wavelengths, in this analysis, were 810 nm, 650 nm, and 610 nm. The same analysis was performed with regard to important wavelengths for determining water content in leaves. It was found that, when the 760 nm wavelength was omitted and the Gaussian process regression algorithm was applied with the 11 other features, the average (after 10 runs) MSE of five-fold cross-validation was calculated to be 22.9037. Similarly, the MSE of the other features was measured as shown in Figure 13. The most significant wavelength for water content determination was found to be 760 nm, followed by 730 nm and 860 nm in descending order. The significance of the other wavelengths was observed to be nearly similar.

## 4. Discussion

In this section, the features of other devices, used in previous works related to N content estimation in leaves, are compared with the proposed multispectral sensor. In several previous publications [3,15,26] that utilized a hyperspectral camera, good correlation around 86%–92% was achieved between images and leaf N content. The other popular devices are Field Spec 3 with $R^{2}$ of 77%–86% [10], imagery from Quick Bird with $R^{2}$ of 79%–83% [12], atLEAF with $R^{2}$ of 76% [41], Green Seeker with $R^{2}$ of 57%–74% [11], Multiplex with $R^{2}$ of 73%–86% [42], and SPAD with $R^{2}$ of 60.21%. All the above-mentioned device accuracies were from in-field experiments, whereas the proposed sensor was tested in a greenhouse-controlled environment. However, most of the devices, except for the hyperspectral camera and Field Spec 3, are based on either chlorophyll (SPAD, Multiplex, atLEAF) or relative greenness (Green Seeker, Quick Bird imagery) instead of N. Moreover, the proposed sensor is useful in estimating N as it operates on more features (12 wavelengths) than SPAD (two wavelengths), atLEAF (two wavelength), Green Seeker (two wavelengths), and Multiplex (four wavelengths). Although the hyperspectral camera and Field Spec are the most accurate in N determination, these devices are very expensive (hyper spectral camera ~$15,000–$50,000, Field Spec series ~$10,000–$20000, Green Seeker ~$700, atLEAF ~$250, and SPAD ~$1500–$2500), whereas the total estimated cost of the proposed prototype, including all the components, is $150. By incorporating the manufacturing labor cost and overhead cost, the approximate estimation of the device might be $200. A complete breakdown of the cost is shown in Table 4. However, this is the first version of the prototype, which can be further modified to reduce the cost. For example, without using the Raspberry Pi (control circuit) development board, the control circuit can be implemented in a custom-made printed circuit board (PCB), according to the design requirements. Moreover, the cost of the device will be significantly reduced if it is mass-manufactured. On a different note, the proposed sensor is relatively lightweight (350 g) compared to the other devices (hyperspectral camera: 1.3–4.5 kg, FieldSpec Series: 5.4 kg). Furthermore, the technologies like hyperspectral camera and satellite imagery are normally used in developed countries, whereas, for farmers from low-resource countries, these expensive devices are out of the question. Additionally, as the device is cheap and portable, it can be manufactured in large quantities and deployed in sensor arrays more flexibly, which unfeasible for expensive and heavyweight devices. It can also be mounted to custom IoT (Internet of things) platforms for wireless monitoring of the N level. Thus, the proposed sensor is very effective as a low-cost, portable, quick, and lightweight device for monitoring N content.

**Figure 14:**
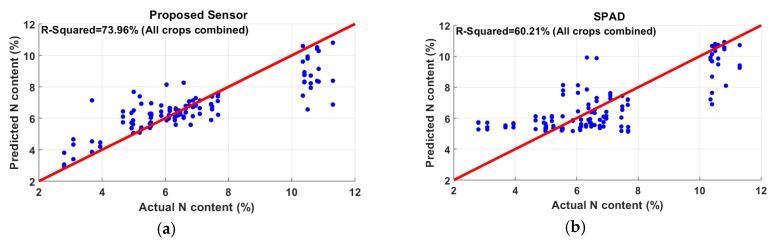
Comparison between (**a**) proposed sensor and (**b**) soil plant analysis development (SPAD). The proposed sensor showed a better correlation (73.96%) with N than SPAD (60.21%).

SPAD is a commercially used meter for indirect measurement of N. In this work, we also took measurements using SPAD. The motivation for performing this analysis was to see how profoundly SPAD readings correlate with N compared to the proposed MS sensor. For this analysis, the same methodology including preprocessing, modeling, and five-fold cross-validation was applied. It is to be noted that the dataset used for the comparison was that with the combination of all four types of plants (121 leaves). It was observed that SPAD predicted the N contents with an $R^{2}$ of 60.21%, whereas the proposed sensor had an $R^{2}$ of 73.96% (Figure 14). It is to be noted that the atLeaf chlorophyll meter is another cheap alternative version to the SPAD. The technical difference between the two models is that SPAD uses two wavelengths (650 nm and 940 nm), whereas atLEAF works at 660 nm and 940 nm [9]. These wavelengths (650 nm or 660 nm) are basically sensitive to chlorophyll. In contrast, the proposed sensor works on 11 more wavelengths (450 nm, 500 nm, 550 nm, 570 nm, 600 nm, 610 nm, 680 nm, 730 nm, 760 nm, 810 nm, and 860 nm) in addition to 650 nm. As it adds more features to the statistical learning process, the proposed sensor performs better than SPAD in determining N content in leaves.

In this study, N estimation results were better than those for water estimation in terms of correlation. One of the main reasons is that plant water content is mainly sensitive to thermal or short-wave infrared regions, but the proposed sensor operates in the visible and NIR regions. Hence, more spectral bands in the short-wave infrared regions are perhaps necessary to improve the water correlation results. Moreover, the values for water content were in a small range from 60%–90%, for which the model is not robust enough to exhibit good correlation. Another limitation of this study is that the proposed device was only tested on canola, corn, wheat, and soybean. Furthermore, both the experiments were performed in a greenhouse-controlled environment. Thus, the performance variations, if applied in field settings, need further investigation, because several parameters like wind, crop stage, and dust may affect the reflectance profile.

## 5. Conclusions

Leaf N and water contents are important indicators of plant health. Most of the non-invasive approaches include expensive equipment. In this paper, a low-cost, portable optical sensor was proposed that can be effectively modeled with an appropriate regression algorithm to determine leaf N. Using the multispectral sensor, we correlated leaf reflectance at 12 wavelengths with crop measurements. The five-fold cross-validation results of the GPR model revealed that the best correlation of N was found in soybean ($R^{2}$ of 82.29%) while, for water content, the best correlation was found in corn (68.41%). Overall, this device shows better performance in estimating N than water. After comparing the N estimation result with a commercially used device (SPAD), we found that the proposed multispectral sensor shows a better correlation with N than SPAD ($R^{2}$ of 60.21%). It is worthwhile mentioning that the overall cost of our proposed sensor is $200, which is very cheap compared to other technologies. However, the utility of the proposed device in water content estimation needs further consideration. Additionally, the accuracy of the device can be further improved by experimenting on more samples and making a robust model for N estimation. Future scope includes using this device to correlate with other nutrients, such as P and K. 

## Figures and Tables

**Table 1:** Validation metrics used for regression. The regression results for both experiments are reported based on these metrics.

Validation Parameter	Definition
Root-mean-square error (RMSE)	$\sqrt{\frac{\sum_{i=1}^{N}{\left(y_{ti}-y_{pi}\right)}^{2}}{N}}$
Mean squared error (MSE)	$\frac{\sum_{i=1}^{N}{\left(y_{ti}-y_{pi}\right)}^{2}}{N}$
Mean absolute error (MAE)	$\frac{\sum_{i=1}^{N}\left|\left(y_{ti}-y_{pi}\right)\right|}{N}$
Co-efficient of determination ($R^{2}$)	$1-\frac{\sum_{i=1}^{N}{\left(y_{ti}-y_{pi}\right)}^{2}}{\sum_{i=1}^{N}{\left(\bar{y_{t}}-y_{ti}\right)}^{2}}$

$y_{ti}$ stands for the actual content of the i-th sample (nitrogen/water), $y_{pi}$ stands for the predicted content of the i-th sample (nitrogen/water), $\bar{y_{t}}$ is the average of actual data, and N is the number of test data points in each fold.

**Table 2:** Five-fold cross-validation results of the N experiment. The average of the metrics from the five folds is reported.

Plant Species	$R^{2}(\%)$	RMSE	MAE
Canola	63.91	1.28	0.87
Corn	80.05	0.50	0.31
Soybean	82.29	0.21	0.12
Wheat	63.21	0.57	0.37
All crops combined	73.96	1.13	0.72

**Table 3:** Five-fold cross-validation results of water. The average of the metrics from the five folds is reported.

Plant Species	$R^{2}(\%)$	RMSE	MAE
Canola	18.02	1.06	0.76
Corn	68.41	1.17	0.75
Soybean	46.38	3.50	2.11
Wheat	64.58	1.16	0.85
All crops combined	46.08	3.97	2.75

**Table 4:** Cost of the components of the proposed system. USD—United States dollar.

Device Components	Approximate Cost (USD)
Sensor1	$25
Sensor2	$25
MUX	$20
Control circuit (RP3)	$50
Power bank	$10
Display	$5
Manufacturing	$50
Others	$15
Total	$200
